# New-Onset Hypothyroidism Manifesting As Myxedema Coma: Fighting an Old Enemy

**DOI:** 10.7759/cureus.23881

**Published:** 2022-04-06

**Authors:** Andrea Santos Argueta, Sotirios G Doukas, Roopa Roy

**Affiliations:** 1 Internal Medicine, Saint Peter’s University Hospital, New Brunswick, USA; 2 Department of Forensic Sciences and Laboratory of Toxicology, University of Crete, Medical School, Heraklion, GRC; 3 Department of Medicine, Saint Peter's University Hospital, New Brunswick, USA; 4 Endocrinology, Saint Peter’s University Hospital, New Brunswick, USA

**Keywords:** thyroid hormone, hypothermia, hypothyroidism, myxedema coma, hashimoto’s thyroiditis

## Abstract

Myxedema coma (MC) is a rare manifestation of severe hypothyroidism. This is a true endocrine emergency that may remain unrecognized. We present a case of a 49-year-old man who presented to the emergency department with generalized weakness and confusion. He was found to have low temperature, bradycardia, hypoxia, hypotension, glucose of 59 mg/dL, normal electrolytes, thyroid-stimulating hormone of 154 IU/mL, and free T4 of 0.1 ng/dL. His anti-peroxidase antibody level was 99 IU/mL. Echocardiography revealed a normal ejection fraction and no evidence of pericardial effusion. On the basis of his presentation and laboratory findings, he was diagnosed with MC, intubated, and admitted to the intensive care unit. Thyroid hormone replacement and glucocorticoid treatment were initiated immediately. After the clinical improvement, the patient was extubated. MC is associated with a high mortality rate and requires prompt recognition and treatment. This rare case reminds us that MC might still be the first manifestation of primary hypothyroidism, although considered an “old enemy”.

## Introduction

Myxedema coma (MC) is a rare but life-threatening presentation of long-standing hypothyroidism that often remains unrecognized. It was first reported in 1850 as a result of severe untreated hypothyroidism; since then, an increasing number of cases have been reported [1]. Owing to thyroid hormone testing and treatment availability, the incidence has declined. Although it is difficult to calculate the exact incidence, some authors estimate it to be 0.22 cases per 100,0000 people, per year [2-3]. This condition is more common in women older than 60 years with long-standing hypothyroidism, and its mortality can exceed 60% without medical treatment [4-5]. The presentation usually includes altered mental status, hypoventilation, hypothermia, hypotension, bradycardia, and hypoglycemia, all of which are associated with hemodynamic instability [2]. Given its high mortality rate, aggressive treatment with thyroid hormone replacement therapy should be initiated upon suspicion of this disease [6]. Here, we discuss a rare case of undiagnosed hypothyroidism in a male patient presenting with MC and describe its clinical manifestations and management.

## Case presentation

A 49-year-old man with no medical history presented to the emergency department of our hospital with generalized weakness, confusion, and obtundation. Vital signs at presentation showed a blood pressure of 85/65 mmHg, pulse of 54 beats per minute, respiratory rate of 32 breaths per minute, O_2_ saturation of 78% on room air, and a temperature of 96.4°F. Physical examination revealed generalized edema, a distended abdomen, and decreased deep tendon reflexes with a delayed relaxation phase. No goiter was noted on neck examination.

**Figure 1:**
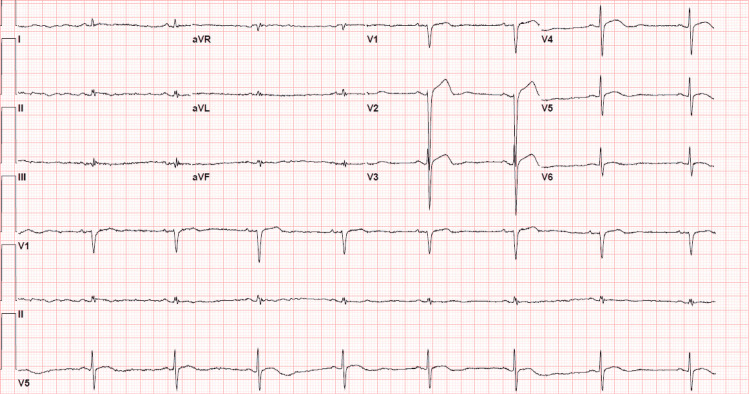
Electrocardiogram

Blood tests showed a low glucose level, normal electrolytes, elevated thyroid-stimulating hormone (TSH), and low free T4 (Table 1). EKG (Figure 1) showed sinus bradycardia with a heart rate of 49 beats per minute and low-voltage QRS. Echocardiography revealed a normal ejection fraction and no evidence of pericardial effusion. Urinalysis revealed a hazy appearance with positive urine nitrites, moderate leukoesterase, 10-20 white blood cells, and 5-10 red blood cells, indicating a possible urinary tract infection (UTI).

**Table 1:** Laboratory Investigations BUN: blood urea nitrogen; TSH: thyroid-stimulating hormone; T4: thyroxine

Laboratory Test	Value	Reference Range
BUN	22 mg/dL	6-20 mg/dL
Creatinine	1.09 mg/dL	0.66-1/26 mg/dL
Glucose	59 mg/dL	74-106 mg/dL
Sodium	145 mmol/L	136-145 mmol/L
Potassium	4.3 mmol/L	3.5-5.1 mmol/L
TSH	154 IU/mL	0.465-4.680 IU/mL
Free T4	0.10 IU/mL	0.78-2.19 IU/mL
Cortisol	>123 mcg/dL	4.5-22.7 mcg/dL
Thyroid peroxidase antibody	99 IU/mL	<1 IU/mL

The patient was admitted to the intensive care unit and, owing to his altered mental status and hypoventilation, was intubated and mechanically ventilated. He received intravenous (IV) levothyroxine, 200 µg once followed by 75 µg daily, and liothyronine 10 µg every 8 h through an orogastric tube as the IV formulation was not available at our institution. An abdominal radiograph series was performed that ruled out bowel obstruction.

Given the hypoglycemia and hypotension, hydrocortisone (100 mg every 8 h) was started. Although cortisol levels were assessed, the results were equivocal owing to ongoing hydrocortisone use, and adrenal insufficiency could not be ruled out. Ceftriaxone 1 g daily was also initiated for suspected UTI. The anti-peroxidase antibody level was 99 IU/mL, indicating a possible underlying Hashimoto’s thyroiditis.

The patient demonstrated gradual and subtle improvements over several days, with progressively increasing T3 and T4 levels. When clinically appropriate, extubation was performed. However, given the recurrent hypercapnic respiratory failure, reintubation was performed. Further optimization of the thyroid medications was performed, and after multiple attempts, the patient was successfully extubated. After extubation and because the patient was hemodynamically stable, hydrocortisone was tapered, and liothyronine was discontinued. Levothyroxine was transitioned from parenteral to oral once he was able to take oral medications and was eventually downgraded to the floor.

## Discussion

MC represents the most extreme form of hypothyroidism, which may progress to death even if diagnosed and promptly treated [5]. According to previous epidemiological studies, myxedema occurs almost exclusively in women older than 60 years [7]. If there is a high suspicion of MC, other possible attributing factors should be investigated, such as current use and discontinuation of thyroid hormone replacement, history of thyroid surgery, radioactive iodine ablation, or history of thyroid dysfunction [5]. Interestingly, our patient was a young man without any history of thyroid disorder or thyroid hormone treatment.

Our patient’s high anti-thyroid peroxidase antibody level indicated Hashimoto’s thyroiditis as the underlying etiology. Similar to our patient, it is estimated that 95% of MC cases occur in patients with primary hypothyroidism, usually due to Hashimoto’s thyroiditis, while central hypothyroidism constitutes only 5% of MC cases [2-4]. Similar to our case, patients with MC due to primary hypothyroidism present with significantly high TSH levels and low T4 levels compared to those with central hypothyroidism, in which both TSH and T4 levels are low [5].

To date, there are no clear diagnostic criteria for MC, but it is associated with multiple organ failure [8]. Low body temperature appears to be a cardinal feature directly associated with mortality and can range from a mild decrease in body temperature to severe hypothermia [2]. The basic pathogenesis is related to decreased thermogenesis due to low intracellular T3 levels, which leads to low body temperature [5]. A passive rewarming process is recommended in patients with MC because active rewarming may lead to peripheral vasodilation and a drop in blood pressure [9].

Other crucial mortality factors in MC include cardiovascular abnormalities that lead to hemodynamic instability and shock [10]. Hypothyroidism commonly manifests as bradycardia, possibly due to alterations in cardiac autonomic inputs [11]. In myxedema, bradycardia, and decreased myocardial contractility, in combination with a reduced preload due to downregulation of T3-regulated liver-derived renin, leads to a significantly decreased cardiac output [12]. Increased peripheral vascular resistance due to reduced endothelial nitric oxide release and decreased cardiac output commonly manifests as narrow pulse pressure [13].

In contrast to chronic hypothyroidism, which commonly presents with diastolic hypertension, MC due to severely dysregulated cardiovascular hemodynamics can present with severe hypotension or shock [14]. A low-voltage EKG might indicate pericardial effusion and suggest prompt echocardiography. However, in patients without pericardial effusion, similar to our case, these EKG findings can be attributed to anasarca. Other non-specific EKG findings in MC include flattened T waves, prolonged QT, and heart blocks [15].

Concomitant adrenal insufficiency could further contribute to hemodynamic instability and shock in patients [16-17]. The underlying cause could be synchronous autoimmune adrenal disease, especially in patients with Hashimoto’s thyroiditis or central disruption of the hypothalamic-pituitary axis [18]. Hyponatremia due to ADH release and hypoglycemia due to downregulated gluconeogenesis can be induced by MC or a concomitant underlying adrenal insufficiency [11-19]. Hemodynamic stabilization with crystalloids, vasopressors, and glucocorticoids is indicated in MC patients with shock [20]. Although in our case, accurate cortisol levels could not be obtained given the emergent administration of hydrocortisone, suspicion of adrenal insufficiency was remarkable as the patient was hypoglycemic and hypotensive at presentation.

In MC, suppression of hypoxic ventilatory drive can be seen secondary to decreased central nervous system sensitivity to hypoxia and hypercapnia [5]. Thyroid hormone therapy for one week may reverse the impaired ventilatory responses in these cases [21]. Persistent respiratory failure may be multifactorial and is associated with decreased respiratory drive, respiratory muscle myopathy, and phrenic nerve neuropathy, which causes diaphragmatic dysfunction [22]. MC can also cause altered mentation owing to electrolyte abnormalities, hypercapnia, or decreased central nervous system excitation [23]. Intubation for airway protection is highly recommended in these patients [9]. As seen in our case, extubation of a patient with MC might be challenging due to poor respiratory drive, and should be approached with optimization of thyroid hormone supplementation.

Other clinical manifestations, such as dry skin, thin hair, hoarseness, non-pitting peripheral edema, macroglossia, and decreased deep tendon reflexes may be present in patients with MC [24]. However, not all patients exhibit full classical findings, making the diagnosis challenging. The presence of myxedema or comma is not pathognomonic and is not required for diagnosis. The presence of other physiological stressors should be considered in any case of suspected MC [21]. A careful review of other potential precipitating factors is recommended, including sedative drugs, cerebrovascular accidents, myocardial infarction, surgery, gastrointestinal bleeding, metabolic disturbances, hypothermia, or even trauma [5]. In our case, urinalysis suggested a UTI, and antibiotic therapy was initiated immediately.

In patients with a high suspicion of MC, thyroid hormone replacement therapy should be initiated immediately while waiting for thyroid function tests. Although thyroid hormone replacement is the mainstay of treatment, there is controversy regarding levothyroxine (T4) monotherapy versus a combination of T4 and liothyronine (T3). Typically, 200-500 mcg of parenteral T4 should be administered as a bolus and then continued at 50-100 mcg daily until the patient could take T4 orally. If available, T3 should be administered at low and gradually increasing doses. It has a 10-20-fold higher affinity to the thyroid hormone nuclear receptor than T4 and could potentially lead to clinical and neurological improvement within 24 hours [25]. However, the use of T3 remains controversial as it was previously associated with higher mortality, possibly due to the precipitation of fatal arrhythmias or myocardial infarction [9-26]. Based on the existing data, some authors recommend using 12.5-25 mcg of T3 every 6 hours, only in severe cases of MC [26-27]. In our case, given the high severity, both T3 and T4 supplementation were used, leading to significant clinical improvement.

Patients with MC may present with ileus, which can interfere with appropriate medication absorption. Bowel edema due to mucopolysaccharide infiltration and neuropathic changes may be present in the MC. As patients may present with ileus, oral therapy may have lower absorption, and an intravenous formulation may be appropriate [27-28]. Unfortunately, the intravenous formulation of T3 was not available in our institution and surrounding institutions, so oral therapy was administered. The response to treatment should be assessed daily or every other day by monitoring TSH and T4 along with clinical cardiovascular, renal, pulmonary, and metabolic improvement [5].

## Conclusions

This rare case reminds us that MC might still be the first manifestation of primary hypothyroidism, although considered an “old enemy.” Our case aims to increase awareness of the clinical manifestations of MC and highlight the importance of assessing thyroid function when clinical suspicion is high, given the high mortality if the diagnosis is delayed or even missed. Also, it suggests that further investigations are necessary to develop effective therapeutic protocols for this devastating endocrinologic emergency.
